# A Joint Saga: Human Leukocyte Antigen (HLA) B27 Ankylosing Spondylitis and Sacroiliitis Association with Henoch-Schönlein Purpura

**DOI:** 10.7759/cureus.39041

**Published:** 2023-05-15

**Authors:** Nicholas D Nassif, Daniel Rosler

**Affiliations:** 1 Internal Medicine, Advocate Aurora Healthcare, Milwaukee, USA; 2 Rheumatology, Advocate Aurora Healthcare, Milwaukee, USA

**Keywords:** henoch-schönlein purpura, ankylosis spondylitis, iga, vasculitis, hla

## Abstract

Henoch-Schönlein purpura (HSP) or IgA vasculitis is a small-vessel vasculitis mediated by IgA deposition, often associated with upper respiratory tract infection and family history. However, there is a rare correlation to human leukocyte antigen (HLA) B27 arthropathy. We present a case of a young boy diagnosed with HSP suffering from arthritis, gait disturbance, and weakness throughout childhood, ultimately diagnosed with ankylosing spondylitis and sacroiliitis clinically, with confirmation through X-ray and supporting HLA B27 testing.

## Introduction

Henoch-Schönlein purpura (HSP) is an IgA-mediated small-vessel vasculitis commonly found in children [1]. HSP often manifests dermatologic, gastrointestinal, musculoskeletal, and renal symptoms. The incidence of HSP is approximately 10-20 cases per 100,000 children per year with most achieving an excellent outcome [1]. Risk factors for HSP include family history and upper respiratory tract infection, especially Group A streptococcus for parvovirus B19. As with most chronic (i.e., genetic) diseases, the goal is to limit disease progression, control associated side effects, and improve quality of life. HSP has a predilection for joint involvement by way of seronegative arthropathy. However, rarely, there has been a coincidental finding of HLA B27 positive axial spondylarthritis [2]. Herein, we describe a case of a young boy with a notable history of HSP who developed persistent lower back pain and uneven gait, ultimately diagnosed with HLA B27 ankylosing spondylitis.

## Case presentation

**Figure 1:**
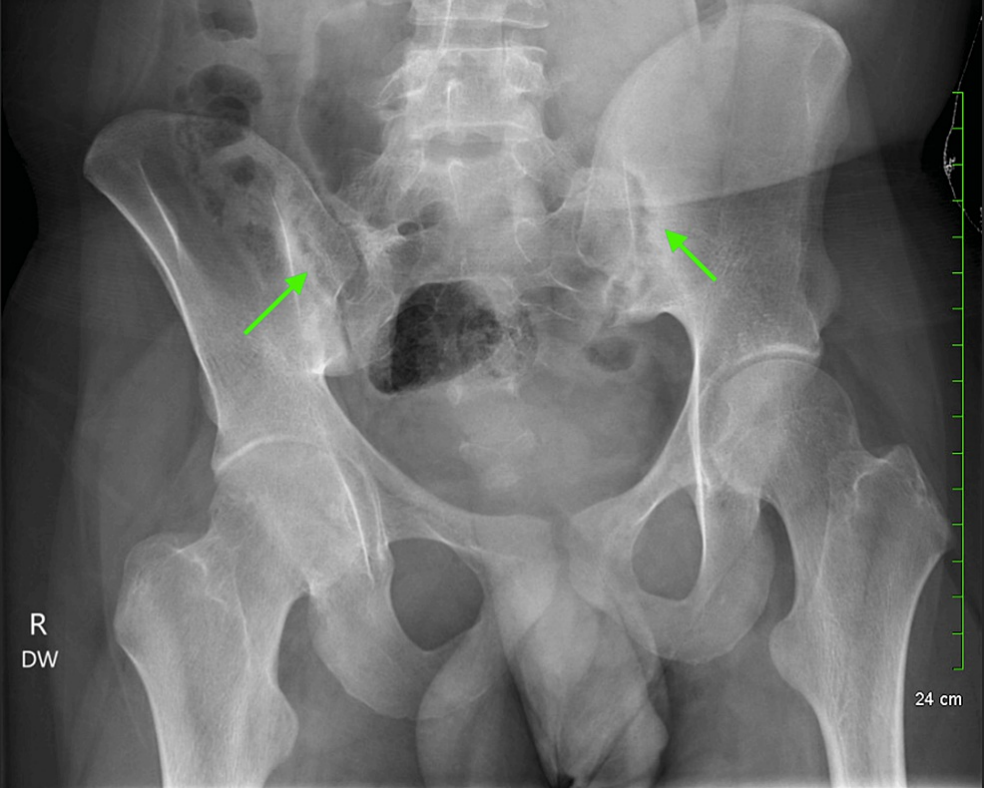
Bilateral hip and sacroiliac joint X-ray demonstrating joint space narrowing with sacroiliac joints showing subchondral lucencies with concern for bilateral inflammatory sacroiliitis.

At 6 years old (2007), our patient visited the primary care physician with concerns of bloody urine, abdominal pain, and skin changes. This appeared to be episodic and at one point had led to hospitalization requiring a six-month course of oral steroids. Biopsy at that time showed mesangial hypercellularity without fibrosis consistent with HSP nephritis. His childhood growth course was otherwise unremarkable. In 2013, he presented to the physician with concerns of arthralgias and myalgias. Inflammatory markers were elevated, as well as rheumatoid factor level. At that time, he did not have overt evidence of arthritis but a few weeks of symptoms. His ASO titers were elevated, documenting a past Group A beta-hemolytic strep (GABHS) infection. The favored diagnosis at that time was reactive arthritis secondary to GABHS. He was maintained on non-steroidal anti-inflammatory drugs (NSAIDs) as well as acetaminophen, and his symptoms were monitored. At 18 years old, he presented to rheumatology with persistent arthralgia, gait disturbance, and weakness. Given a positive family history, HLA B27 was ordered, as well as a lumbosacral X-ray. X-rays documented ankylosing spondylitis with sacroiliitis (Figure 1), and HLA B27 returned positive. He was diagnosed with ankylosing spondylitis and sacroiliitis on a history of HSP and was started on NSAIDs and appropriate immunotherapy, and his symptoms improved.

## Discussion

HSP is a systemic, small-vessel vasculitis mediated by IgA notorious for the clinical triad of palpable purpura, arthralgia, and abdominal pain. Pathogenesis is mediated by IgA immune complex deposition in the small vessels, with deposition causing the physical manifestations of the disease in the skin, gastrointestinal tract, and kidneys (hematuria, proteinuria). While associated conditions include Buerger's disease, or IgA nephropathy, and upper respiratory tract infections, rarely, is HSP associated with HLA B27 [1-2]. In fact, IgA nephropathy has a well-documented history of association with seronegative spondyloarthropathies.

On review, multiple cases of HSP have been associated with positive HLA B27 with notable arthritis and arthralgia. Some studies have outlined an association to HLA-B alleles when compared to controls (increased occurrence of 8.3% compared to the 1.5% occurrence in controls) [3]. This study did not find a direct association to HLA B27, rather, an association between HLA-B*41:02 and HLA-DRB1*01:03 and the development of HSP. There was no difference found between occurrence rates in male and female patients. Su Jin Kim et al. described a case of a 19-year-old male diagnosed with HSP who subsequently developed ankylosing spondylitis with positive HLA B27 testing [4]. John et al. described a case of a 26-year-old male who had abdominal pain and palpable purpura with radiography demonstrating sacroiliitis; subsequent workup was positive for HSP and HLA B27 [2]. Finally, Jung et al. described a unique case of a 59-year-old male with a known history of ankylosing spondylitis treated with TNF-α inhibitor (Infliximab) for approximately 60 months, who ultimately developed palpable purpura and abdominal pain; radiography demonstrated small-bowel wall thickening with biopsies showing leukocytoclastic vasculitis (LSV) positive for HSP that resolved with steroid therapy [5]. 

One similar but differentiable diagnosis is LSV. Histology demonstrates neutrophil infiltration into the vessel wall with depositions of IgA, IgG, IgM, and C3, with a strong link to patients diagnosed with SLE [6]. Biopsies of HSP will generally show only IgA mesangial deposition. While HSP may have significant extracutaneous manifestations as described above, these are far less common in LSV [6]. Finally, while LSV can affect any patient of any age, we generally see a slight male youth predominance in HSP, as can be seen in our case presentation [6].

Our goal is to bring to light the benefits of early recognition of arthritis in patients with a history of HSP not as a standalone diagnosis but rather as part of an associated clinical syndrome. We hope to encourage future documentation of patients with both diagnoses in the hopes of presenting a unified syndrome. In doing so, we may be able to prevent the progression of axial spondyloarthropathy and the severe debility our patients endure.

## Conclusions

In conclusion, indolent musculoskeletal complaints in a patient with a previous diagnosis of HSP can be a subtle clue into the possible underlying diagnosis of HLA B27 positive ankylosing spondylitis. While correlation does not imply causation, we review several cases of young men with a previous diagnosis of HSP with an ultimate diagnosis of ankylosing spondylitis. Furthermore, we discuss the case of a male treated with immunotherapy for ankylosing spondylitis that may have triggered the development of HSP, possibly elucidating an underlying connection between the two diagnoses. When HSP is diagnosed, routine musculoskeletal examination, radiography of the sacroiliac spine, and appropriate use of HLA B27 testing should be considered.
